# Mapping the *Pax6* 3’ untranslated region microRNA regulatory landscape

**DOI:** 10.1186/s12864-018-5212-x

**Published:** 2018-11-15

**Authors:** Bridget C. Ryan, Kieran Lowe, Laura Hanson, Talveen Gil, Lauren Braun, Perry L. Howard, Robert L. Chow

**Affiliations:** 1 Department of Biology, University of Victoria, Victoria, BC V8W 3N5 Canada; 2 Department of Biochemistry and Microbiology, University of Victoria, Victoria, BC V8W 2Y2 Canada

**Keywords:** Gene regulation, Pax6, microRNA, Bioinformatics, Expression profiling, Pull-down assays

## Abstract

**Background:**

PAX6 is a homeodomain transcription factor that acts in a highly dosage-sensitive manner to regulate the development and function of the eyes, nose, central nervous system, gut, and endocrine pancreas. Several individual microRNAs (miRNA) have been implicated in regulating PAX6 in different cellular contexts, but a more general view of how they contribute to the fine-tuning and homeostasis of PAX6 is poorly understood.

**Results:**

Here, a comprehensive analysis of the *Pax6* 3′ untranslated region was performed to map potential miRNA recognition elements and served as a backdrop for miRNA expression profiling experiments to identify potential cell/tissue-specific miRNA codes. *Pax6* 3’UTR pull-down studies identified a cohort of miRNA interactors in pancreatic αTC1–6 cells that, based on the spacing of their recognition sites in the *Pax6* 3’UTR, revealed 3 clusters where cooperative miRNA regulation may occur. Some of these interacting miRNAs have been implicated in α cell function but have not previously been linked to Pax6 function and may therefore represent novel PAX6 regulators.

**Conclusions:**

These findings reveal a regulatory landscape upon which miRNAs may participate in the developmental control, fine-tuning and/or homeostasis of PAX6 levels.

**Electronic supplementary material:**

The online version of this article (10.1186/s12864-018-5212-x) contains supplementary material, which is available to authorized users.

## Background

PAX6 is a highly conserved transcription factor that is expressed in a specific spatio-temporal pattern in several tissues during vertebrate development, and plays an important role in cell fate determination and tissue differentiation [1]. *Pax6* is expressed in the developing retina, lens and cornea, and continues to be expressed in several mature ocular cell types [2–6]. *Pax6* is also expressed in the developing and mature endocrine pancreas [7, 8], central nervous system (CNS), and olfactory system [2, 3, 9], gut [10] and osteocytes [11]. In the complete absence of *Pax6*, eyes and nasal structures fail to develop [12–14], and patterning in the forebrain and specification of hormone-producing cells in the endocrine pancreas are severely perturbed [7, 8, 15]. *Pax6* is also required for maintenance of the progenitor cell pool in the cortex and spinal cord [16, 17] and in the retina for progenitor cell multipotency [18].

PAX6 function is particularly sensitive to dosage: too little or too much PAX6 can have profound effects on tissue development and maintenance. The requirement for precise PAX6 dose is exemplified by the semi-dominant phenotypes associated with PAX6 haploinsufficiency and from overexpression phenotypes. Loss of a single copy of *Pax6/PAX6* results in a small eye phenotype in rodents [12–14] and is the primary cause of the poly-symptomatic and progressive disease aniridia in humans [1, 19, 20]. Though *Pax6/PAX6* haploinsufficiency is not associated with overt defects in pancreatic development, mice lacking one copy of *Pax6* have impaired proinsulin processing and glucose metabolism [21]. In humans, *PAX6* heterozygosity is associated with glucose intolerance [22]. *Pax6* overexpression in mice carrying multiple copies of the human *PAX6* gene impairs normal development of the eye, leading to reduced eye size and photoreceptor loss in the retina [23] and causes cell autonomous defects in late cortical progenitor proliferation, resulting in decreased thickness of superficial cortical layers [24]. Transgenic mice overexpressing *Pax6* during early pancreas development display perturbed development of the endocrine pancreas, β-cell apoptosis, and impaired glucose stimulated insulin secretion [25]. A few cases of *PAX6* gene duplication in humans have been reported, in which a band of chromosome 11, including *PAX6*, *WT1* and *ELP4* genes, was duplicated causing mild ocular defects and mental retardation [26, 27], suggesting that increased *PAX6* dosage in humans may be also deleterious. However, the physiological mechanism(s) regulating precise PAX6 expression levels have not been elucidated.

Post-transcriptional regulation of *Pax6* by miRNAs may represent an important mechanism for maintaining the correct dosage of Pax6. MicroRNAs are 21–25 nucleotide non-coding RNAs that complementary base pair to 6–8 nucleotide target sites usually located within messenger RNA (mRNA) 3′ untranslated regions (3’UTRs) via seed sequences located at their 5′ ends [28, 29]. MiRNAs act post-transcriptionally as sequence-specific guides that recruit silencing complexes to target transcripts and either repress translation or promote increased mRNA turnover [30, 31]. Since the repressive effect of miRNAs on protein expression from targeted transcripts is relatively small is it thought that they function primarily to fine-tune protein translation [32, 33]. Regulation of an individual target transcript can be influenced by the cooperative activity of multiple miRNAs, acting through multiple target sites. For example, closely spaced miRNA target sites can act synergistically [34–36], multiple miRNAs can simultaneously bind [37] and cooperatively regulate a single target transcript [38–40], and transcription factors and developmental genes are enriched among genes predicted to be targeted by multiple miRNAs [34, 41].

Several miRNAs have been implicated as direct regulators of *Pax6* during cell fate specification and boundary formation. In vitro work in mouse embryonic stem cells suggests that the miR-290 family of miRNAs facilitate early mesendoderm lineage specification by targeting the pro-ectodermal *Pax6* [42]. During in vitro differentiation of human embryonic stem cells into neuroectodermal and epidermal cells, the miR-96 family of miRNAs may target *PAX6* in epidermal-fated ectodermal cells and help sharpen the boundary between cells fated to become epidermis versus neuroectoderm [43]. miR-450b-5p may regulate *Pax6* during eye development to prevent ocular commitment of presumptive eyelid epidermis and sharpen the boundary between PAX6-positive corneal epithelium and PAX6-negative epidermis [44]. The highly conserved neuroendocrine microRNA, miR-7, is an important regulator of *Pax6* in the pancreas and brain. Human *PAX6* transcript is a target of miR-7 and can be regulated through two miR-7 target sites in the 3’UTR [45]. During mouse pancreatic development, regulation of *Pax6* by miR-7 may refine the proportion of endocrine cell types formed [46]. In the adult mouse SVZ, miR-7 may function to maintain a steep dorsal-ventral gradient of PAX6 expression and refine the proportion of dopaminergic periglomerular neurons (PGNs) formed in the olfactory bulb [47]. Finally, miR-7 represses oligodendrogenesis and promotes neurogenesis in vitro by targeting *Pax6* [48].

Here, we performed a comprehensive analysis of the *Pax6* 3′ untranslated region to identify potential miRNA recognition elements. Using miRNA expression profiling experiments, we identified potential cell/tissue-specific miRNA codes. Finally, *Pax6* 3’UTR pull-down studies were used to identify a cohort of miRNA interactors in pancreatic αTC1–6 cells which, based on the spacing of their recognition sites in the *Pax6* 3’UTR, revealed 3 clusters where cooperative miRNA regulation may be occurring. Our findings define the functional landscape of miRNA regulation of PAX6 expression.

## Methods

### MRE prediction and selection

“Probability of Interaction by Target Site Accessibility” (PITA) was used to identify all miRNAs predicted to target 876 bp of the mouse *Pax6* 3’UTR [49]. The miRNA target site prediction tools TargetScan [50], MicroCosm [51], Diana-microT [52] and miRanda [53] were also used. (See Additional file 1: Table S1 for full list of miRNAs selected for profiling). Only 7mer-m8, 7mer-A1 and 8mer MREs with or without a single G:U “wobble” pair, and 6mer MREs without a single G:U pair were considered for further analysis. The ImiRP target site prediction tool [54] was used to validate MRE type of PITA predictions. Multiz alignment of 60 vertebrate *Pax6* 3’UTR sequences available through the University of California Santa Cruz Genome Browser [55] was used to access MRE conservation, and miRBase was used to determine the organisms in which miRNAs of interested are expressed.

### Animals

All research on mice was performed with approval of the University of Victoria Animal Care Committee in compliance with the Canadian Council on Animal Care (CCAC) guidelines for the ethical treatment of research animals. All experiments in this study were performed on 129S1 mice (strain #002448, The Jackson Laboratory, Bar Harbor, ME).

### Tissue harvesting and RNA isolation

Adult tissues were harvested from male and female 2-month-old 129S1. For harvest of embryonic retina, 129S1 pregnant dams were euthanized (5% isoflurane followed by cervical dislocation) 12 days post-coitus (E12.5). All tissues were dissected in ice-cold phosphate buffered saline (PBS).

To isolate embryonic day 12.5 retinal tissue from other ocular tissues, developing retinas were first separated from retina pigment epithelium (RPE) in PBS containing 500 units/ml DNase I (ThermoFisher, 18,047,019) and then incubated for 10 min in Hank’s Balanced Salt Solution (ThermoFisher, 14,025,092) containing 0.8 units/ml Dispase II (Roche, 04942078001) with 5% carbon dioxide prior to dissection of the lens. All E12.5 retinas from a single litter were pooled, and three individual litters were collected.

All dissected tissues were put into 1 ml TRIzol (ThermoFisher, 15,596–018) in tissue homogenizing tubes (Precellys, BER-KT0396110092) and homogenized for 1 min at 3000 RPM using a digital disruptor genie (Scientific Industries, SI-DD38). Total RNA isolation was carried out according to manufacturer’s protocol and RNA was resuspended in nuclease-free water and quantitated using a NanoDrop ND Spectrophotometer.

### 3’RACE (rapid amplification of cDNA ends)

3’ RACE was performed based on a previously described protocol [56]. Briefly, Reverse transcription using the GeneRacer kit (Invitrogen) was carried out on 1 μg of total RNA using the Anchored PolyT Reverse primer to prime cDNA synthesis. Nested amplification of cDNA ends was performed using Q5 High-Fidelity enzyme (New England BioLabs) using primer combinations F1 + R1 followed by nested primers F2 + R2 to amplify the *Pax6* 3’UTR, or F3 + R1 followed by F4 + R2 to amplify the reverse orientation transcript. The first amplification steps were carried out as follows: one 2-min denaturation cycle at 94 °C followed by 30 cycles consisting of 10 s at 98 °C, 30 s at 70 °C, 2 min at 72 °C; and a final extension cycle for 5 min at 72 °C. The nested amplification steps were carried out using the following conditions: one 2-min denaturation cycle at 94 °C followed by 30 cycles consisting of 10 s at 98 °C followed by 2.5 min at 72 °C; and a final extension cycle for 5 min at 72 °C.

#### 3’ RACE primers

Anchored PolyT Reverse: 5’-GCTCGCGAGCGCGTTTAAACGCGCACGCGTTTTTTTTTTTTTTTTTTVN-3′.

R1: 5’-GCTCGCGAGCGCGTTTAAAC-3′.

R2: 5’-GCGTTTAAACGCGCACGCGT-3’

F1: 5’-TGTCCTGAACTGGAGCCCGGGAATGGA-3’

F2: 5’-GGACCTTTGCGTACAGAAGGCACGGTAT-3’

F3: 5′- TAATCTAGGCCAGGACC-3’

F4: 5’-TTCCTAGTGAATCCCTTGTTGC-3′

### RNA sequencing

Pre-enrichment of polyA RNA, standard sequencing library/sample preparation, and Illumina sequencing was performed by LC Sciences (Houston, TX, USA).

### MS2-MBP and MS2 binding site plasmids

The expression vector expressing the MS2-MBP fusion protein was a gift from Melissa Moore (University of Massachusetts, Worcester). The fusion protein was purified as described in Jurica et al. (2002) [57].

890 bp of the mouse *Pax6* 3’UTR followed by the SV40 early polyadenylation signal and flanked by Spe1 and Afl2 restriction sites was synthesized by BioBasic Inc., and cloned downstream of the Turbo Green Fluorescent Protein (TurboGFP) gene in the pCMV-TurboGFP-dest1 plasmid vector (Evrogen, FP519) using Xba1 and Afl2 restriction sites. The Q5 site-directed mutagenesis kit (NEB, E0554S) was used to introduce a 2nucleotide substitution into the miR-375 MRE located at *Pax6* 3’UTR position 201 in the TurboGFP-dest1–3’UTR plasmid. The primers used for miR-375 MRE mutation were F: TATCAGTTGGggCAAATCTTCATTTTGGTATCCAAAC and R: CCGTGCCTTCTGTACGCA, where lowercase letters indicate mutated sequences. We designed a sequence containing a tandem array of three MS2 binding sites, ACATGAGGATCACCCATGT, interspersed by 17 random nucleotides [58, 59]. The Q5 site-directed mutagenesis kit was used to introduce the MS2 binding sequence into TurboGFP-dest1 and TurboGFP-dest1–3’UTR plasmids using the following primers: Common forward: AGGATCACCCATGTCTCGGGAGTACCAGAGAACATGAGGATCACCCATGTAG-GTCCGTCATAATCAGCCATACCACA; TurboGFP-dest1-specific reverse: CATGTCTTTATCATGACG-AAGTACATGGGTGATCCTCATGTTTGACATGCTCTAGAGTCGCGGCCGATCC; TurboGFP-dest1–3’UTR-specific reverse: CATGTCTTTATCATGACGAAGTACATGGGTGATCCTCATGTTTGACATGCAG-GTTTAAAACTCTTGCAAG.

### TurboGFP qPCR primer design and efficiency

Primer-BLAST [60] was used to design quantitative PCR (qPCR) primers for quantification of TurboGFP transcript, with an amplicon size of 100–150 nucleotides. Selected primers had the sequences F: CCCGCATCGAGAAGTACGAG, R: GCGGATGATCTTGTCGGTGA. Primer pair efficiency was calculated using a ten-fold dilution series of cDNA prepared from TurboGFP-transfected αTC1–6 (ATCC CRL-2934) cell lysate. Cycle threshold (Ct) values were plotted versus dilution factors in a base-10 semi-logarithmic graph, the correlation coefficient was confirmed to be greater than 0.99, and amplification efficiency was calculated as 10^(1/slope)^. Primer efficiency was found to be 1.952.

### Cell culturing and transfection

Mouse transgenic pancreatic alpha cells (αTC1–6, ATCC CRL-2934) were cultured in DMEM (low glucose, pyruvate, ThermoFisher, 31,600–034) supplemented with 10% FBS (Life Technologies, 16,000–044), 15 mM HEPES, 0.1 mM non-essential amino acids (ThermoFisher, 11,140–050), 0.02% BSA (Sigma-Aldrich, A7906-50G), 1.5 g/L sodium bicarbonate, and 2.0 g/L glucose. Mouse transgenic pancreatic beta cells (βTC-6, ATCC CRL-11506) were cultured in DMEM (ThermoFisher, 11,960–044) supplemented with 4 mM L-glutamine (ThermoFisher, 25,030), 1 mM sodium pyruvate (ThermoFisher, 11,360), and 15% FBS. Both cell types were cultured at 37 °C with 5% carbon dioxide. For total RNA harvest, cells were grown to approximately 80% confluence in 100 mm culture dishes, washed once with PBS, and lysed with 1 ml TRIzol.

αTC1–6 cells were transfected in 6-well dishes with TurboGFP MS2 binding site plasmids 24 h post-seeding, using jetPRIME transfection reagent (Polyplus, 114–07), following the manufacturer’s protocol. Each well was transfected with 3μg of plasmid DNA and 6 μl jetPRIME reagent in 200 μl jetPRIME buffer. 48 h post-transfection, cells were washed with PBS and lysed using a non-denaturing lysis buffer containing 20 mM Tris pH 7.5, 200 mM NaCl, 2.5 mM MgCl_2_, 0.05% IGEPAL, 60 U/ml Superase-In (Ambion, AM2696), 1 mM DTT, and Complete protease inhibitor (Roche, 04693124001).

### miTRAP

miTRAP protocol was performed as described in [37]. Cell lysates were briefly incubated on ice and supernatant was removed following centrifugation. 25 μl magnetic amylose beads (NEB, E8035S) per sample were blocked in lysis buffer containing 0.2 μg/μl yeast tRNA (Invitrogen, 15,401–029) and 0.2 μg/μl BSA (Ambion, AM2616), and then bound with 2.5 μg MS2-MBP protein. Supernatants were incubated with MS2-MBP bound beads for 3 h at 4 °C. Beads were washed and resuspended in 50 μl lysis buffer and transferred to 1 ml TRIzol LS (ThermoFisher, 10,296,028) and RNA was isolated according to manufacturer’s protocol. 1 μl RNA grade glycogen (ThermoFisher, R0551) was added to the aqueous phase of miTRAP products to improve RNA precipitation.

### cDNA preparation

15 μl reverse transcriptase (RT) reactions were prepared for miRNA RT-qPCRs. For miRNA profiling experiments and miTRAP experiments using TaqMan multiplex qPCR arrays, 750 ng and 60 ng RNA, respectively, was used per RT reaction. For miTRAP experiments using individual small RNA TaqMan assays for miR-375, 22.5 ng RNA was used per RT reaction. RT reactions were prepared using TaqMan MicroRNA Reverse Transcription kit (ThermoFisher, 4,366,596). A custom RT primer pool containing primers specific for the 95 selected miRNAs (ThermoFisher, 4,449,141) was used to prepare cDNA for TaqMan multiplex arrays, and an individual RT primer specific for miR-375 (ThermoFisher, 4,427,975, 000564) was used to prepare cDNA for individual miRNA qPCRs. The RT reactions were run following the manufacturer’s protocols for custom TaqMan array miRNA cards with preamplification and for TaqMan small RNA assays.

TurboGFP cDNA synthesis was performed in 20 μl reactions using the Quantitect reverse transcription kit (Qiagen, 205,311) following the manufacturer’s protocols. 60 ng and 100 ng RNA per reaction were used to prepare TurboGFP cDNA for normalizing results from TaqMan multiplex arrays and individual miRNA qPCRs, respectively.

### Quantitative PCR

miTRAP cDNA for use with TaqMan miRNA multiplex arrays was first preamplified using TaqMan PreAmp Master Mix (ThermoFisher, 4,391,128) and custom miRNA PreAmp primer pool. The qPCR reactions for miRNA profiling experiments and miTRAP experiments were prepared using TaqMan Universal Master Mix II with UNG (ThermoFisher, 4,440,038), and custom miRNA microfluidic cards were run on an Applied Biosystems 7900 HT Fast Real Time PCR System fitted with the 384-well block. qPCR reactions for miR-375 were prepared using Universal Master Mix II with UNG and TaqMan small RNA assay for miR-375 (ThermoFisher, 4,427,975, 000564), and were run in MicroAmp fast 96-well reaction plates (0.1 ml, Applied Biosystems, 4,346,907) covered with optical adhesive covers (Applied Biosystems, 4,360,954) using the 7900 HT Fast Real Time PCR System fitted with the 96-well block. All protocols were performed following the manufacturer’s instructions.

qPCR reactions for TurboGFP were performed using QuantiTect SYBR Green PCR kit (204143) and run using the Stratagene Mx300P qPCR system (Agilent Genomics). PCR reaction settings were as follows: hot start for 15 min at 95 °C, and amplification 15 s at 95 °C, 30 s at 60 °C, 30 s at 72 °C repeated for 40 cycles with data recorded twice during the extension step.

### Data analysis

For miRNA tissue profiling analysis, three independent samples were collected and a miRNA was only considered to be expressed if it had a Ct value of less than 40 in all three samples. Levels of each miRNA were calculated relative to U6 snRNA by ΔCt = Ct(U6) – Ct(miRNA), and Relative miRNA level = 2^ΔCt^.

For miTRAP experiments, any samples for which Ct(no RT control) – Ct(+RT) was less than 10 were excluded from the analysis. Except for qPCR reactions performed using the TaqMan multiplex array cards, all qPCR reactions were performed in triplicate. If the standard deviation of the Ct value between triplicate technical replicates was greater than 0.5, the sample was discarded from the analysis. miTRAP experiments using multiplex array cards were performed in quadruplicate, and miRNAs were only considered detected if the Ct values for purification with the wild type *Pax6* 3’UTR were less than 40 in all four replicates. If any Ct values for GFP-MS2 control purifications were undefined (i.e. greater than 40), but the miRNA was detected in all four purifications with the wild type *Pax6* 3’UTR, the undefined Ct was defined as 40.

Relative GFP expression for TurboGFP affinity purification analysis with and without the MS2 binding sites was calculated using Pfaffl’s method [61] without a reference gene, where ΔCt = Ct[GFP] – Ct[GFP-MS2] and GFP Fold difference = 1.952^ΔCt^. Analysis of miR-375 purification with and without transfection of TurboGFP-MS2 was performed using the comparative Ct method without normalization to an internal control, where ΔCt = Ct[Untransfected] – Ct[GFP-MS2] and miR-375 Fold difference = 2^ΔCt^. Normalized relative miR-375 quantity (NRQ) with the wild type and miR-375 MRE mutant *Pax6* 3’UTRs was performed using Pfaffl’s method with qBase+ software [62]. NRQ was calculated by ΔCt = Ct[GFP-MS2] – Ct[GFP-WT3’UTR-MS2] or ΔCt = Ct[WT3’UTR-MS2] - Ct[375MUT3’UTR-MS2], and NRQ = 2^ΔCt[miRNA]^/1.952^ΔCt[GFP]^. Mann-Whitney U test was used for statistical analysis of qPCR data.

miTRAP ratio was calculated by dividing the relative miRNA abundance from the *Pax6* 3’UTR pull-down by the relative miRNA abundance in αTC1–6 cell lysate. Mean Ct for each miRNA from the four *Pax6* 3’UTR pull-down replicates was calculated. Relative miRNA abundance with the *Pax6* 3’UTR was calculated by 1.952^mean Ct[GFP]^/2^mean Ct [miRNA}^.

## Results and discussion

### Characterization of *Pax6* 3’UTR length

**Fig. 1:**
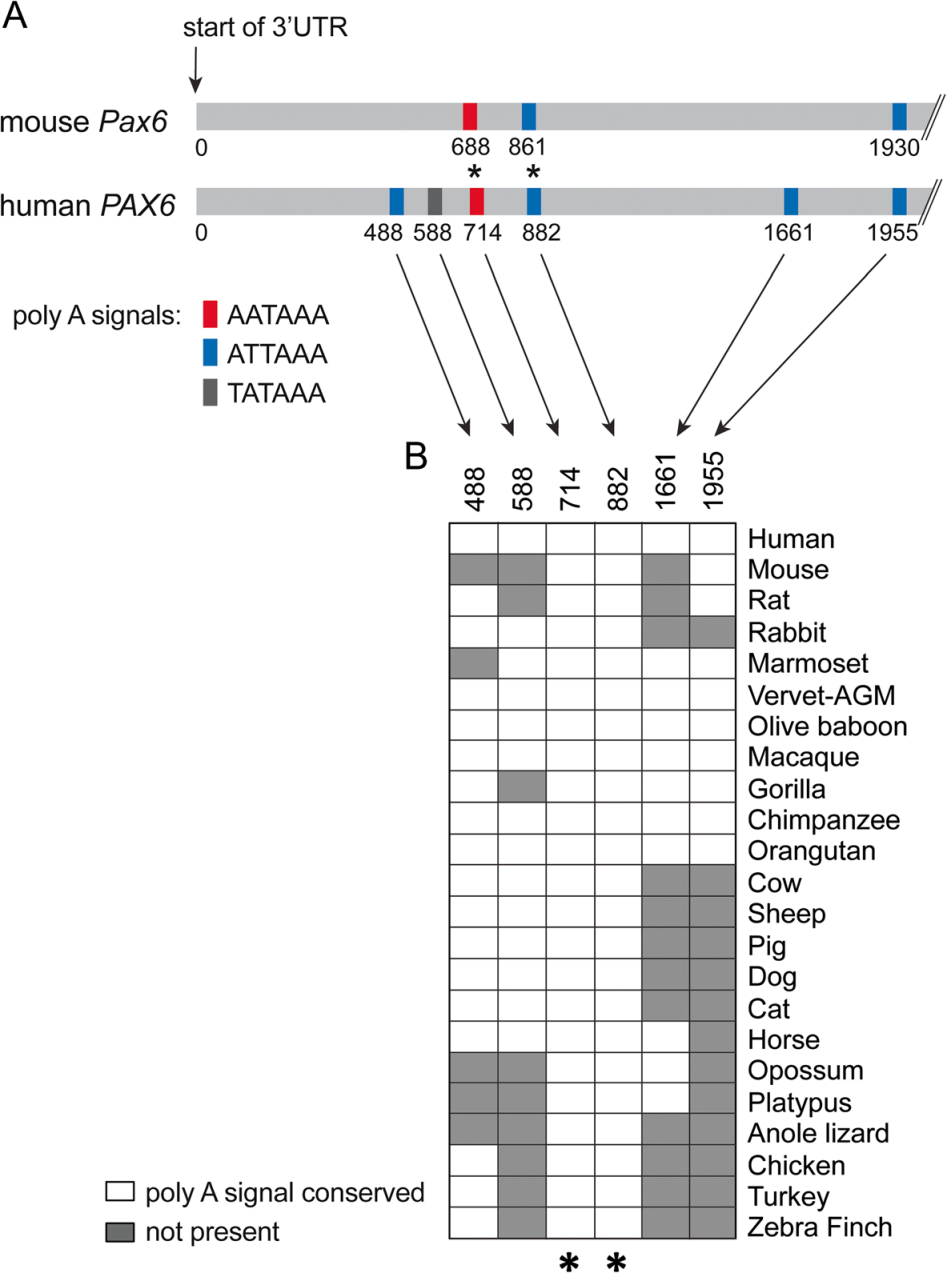
Predicted vertebrate *Pax6* polyadenylation signals and conservation. **a** Location of putative polyadenylation signals (AATAAA, ATTAAA, TATAAA) in the mouse and human *Pax6*/*PAX6* genomic region downstream of the stop codon. Neither mouse nor human *Pax6*/*PAX6* have a AGTAAA polyadenylation signal within the region indicated. **b** Table showing conservation of the human *PAX6* polyadenylation signals across amniotes. Asterisks in (**a**) and (**b**) indicate two polyadenylation signals that are conserved in all of the species examined

As a first step to determine how microRNAs regulate the expression of *Pax6*, we sought to identify a region that best represents the *Pax6* 3’UTR. The initial characterization of the mouse *Pax6* mRNA [9] identified a 3’UTR that was 1008 nucleotides long, however, the absence of a poly(A) signal at the end of the sequence made it unclear whether this represented the complete 3’UTR region. There are three putative polyadenylation (poly(A)) signals within the first 2000 nucleotides downstream of the mouse *Pax6* stop codon at positions 688, 861 and 1930 (Fig. 1a). These three poly(A) signals are conserved at positions 714, 882 and 1955 in the human *PAX6* 3’UTR. Human *PAX6* encodes three additional poly(A) signals at nucleotide positions 488, 588 and 1661. Among the six human *PAX6* poly(A) signals, only those at positions 714 and 882 are conserved in all of the 23 amniote species examined (Fig. 1b), making them good candidates for functional poly(A) signals in vivo.

**Fig. 2:**
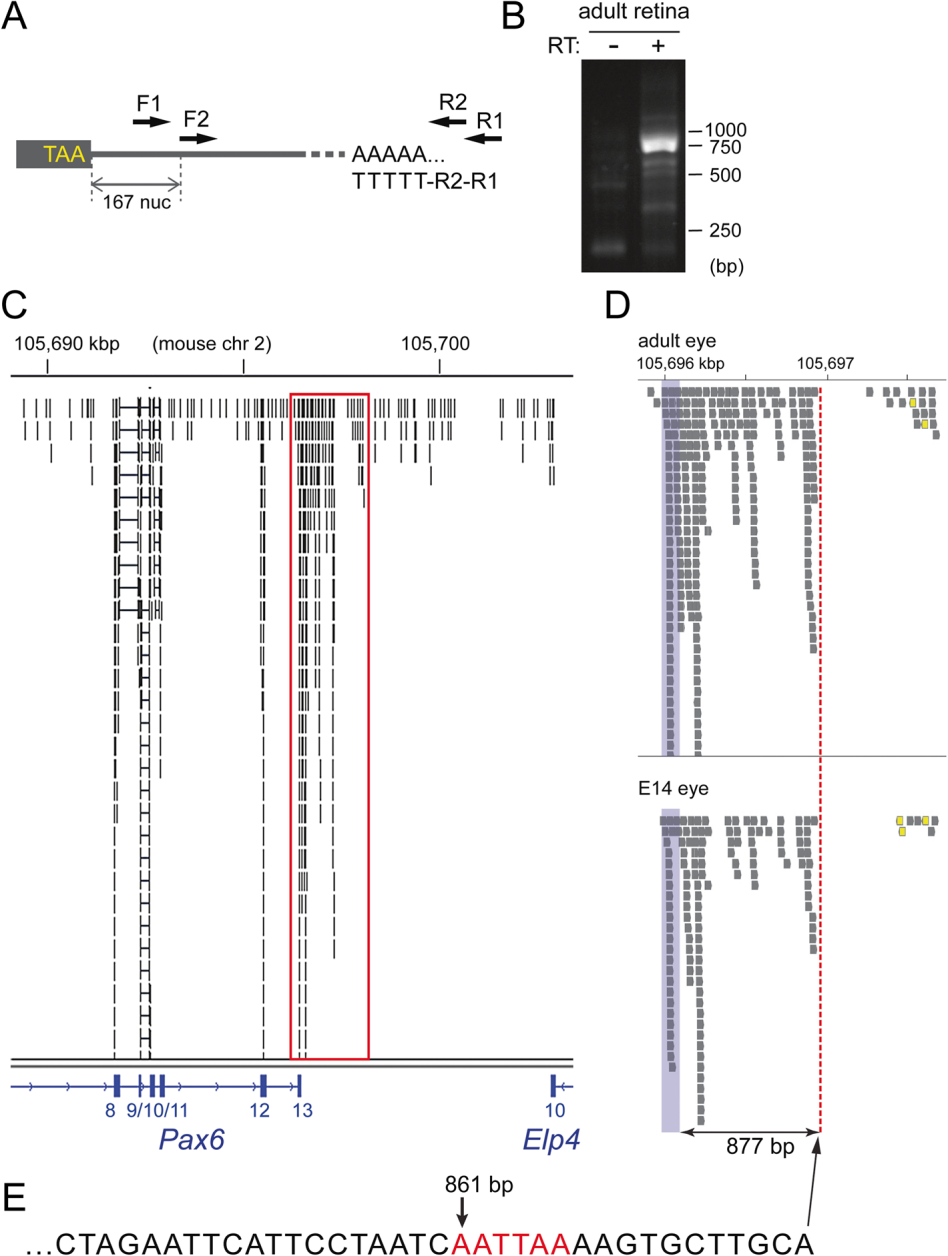
Characterization of the mouse *Pax6* mRNA 3′ terminus. **a** Amplification strategy used in 3’ RACE approach to identify the *Pax6* mRNA 3′ end. “TAA” represents the Pax6 stop codon. Nested primers F1 and F2 were used in combination with reverse primers (R1, R2) built into the poly-T primer used to generate cDNA. **b** 3’ RACE performed on adult retina total RNA. A predominant band was observed at 700–750 bp in contrast to minus reverse transcriptase (− RT) negative control. Weaker bands above and below the predominant 700-750 bp band were also observed. **c** Primary read data from RNA-seq experiment performed on polyadenylation-selected adult mouse eye mRNA. Reads were superimposed onto the 3′ end of the *Pax6* region containing exons 8–13 and the 3′ end of the adjacent gene *Elp4*. The box encompassing *Pax6* exon 13 is shown at a higher magnification in (D – top plot). The light blue shaded regions in (**d**) indicate the end of the *Pax6* coding region. The dotted line indicates the most 3′ end point for Pax6 in both adult eye (D, top plot) and embryonic day 14 (E14) retina (D, bottom plot). **e** Sequence read corresponding to the most 3′ read indicated by the dotted line in (**d**). The putative polyadenylation signal located at position 861 of the *Pax6* 3’ UTR is highlighted in red

Two experimental approaches were next used to determine the length of *Pax6* 3’UTR. First, 3′ rapid amplification of cDNA ends (3’ RACE) was performed on adult mouse retinal total RNA (Fig. 2a, b). This approach yielded a major band, which based on our experimental design (Fig. 2a), corresponds to a 3’UTR of 877 nucleotides. In addition to this major band, a number of weaker bands, both smaller and larger, were observed (Fig. 2b). Subcloning and sequencing revealed that many of the 3’RACE products terminated after the poly(A) sequence at nucleotide position 861 of the 3’UTR (data not shown).

As a second approach, RNA-seq was performed on adult eye poly(A)-selected RNA (Fig. 2c, d, top plot). Similar to the 3’RACE results, RNA-seq reads revealed a Pax6 3’UTR endpoint positioned in close proximity to the 861 poly(A) sequence (Fig. 2d, red dashed vertical line). A “CA” positioned at the end of this region at nucleotide 870 (Fig. 2e) and 17 nucleotides upstream of a 16 nucleotide stretch that is 87.5% U/G is a good candidate for functioning in transcript cleavage. CA sequences facilitate transcript cleavage when positioned 15–30 nucleotides downstream of the poly(A) signal and ~ 20 nucleotides upstream of a U/G rich region [63]. Since 3’UTR length can be differentially regulated between developmental and adult stages [64, 65] RNA-seq was also performed on poly(A)-selected RNA from embryonic day 14 eye. Similar to the adult eye, RNA-seq reads from embryonic eye cDNA indicate that *Pax6* mRNA utilizes the poly(A) signal at nucleotide 861 (Fig. 2d, lower panel).

Interestingly, in both adult and embryonic eye tissues, sequence reads in reverse orientation to *Pax6* were observed immediately downstream of the 861 poly(A) signal (Fig. 2d, yellow colored reads). 3’ RACE revealed a reverse transcript that is polyadenylated and utilizes a robust poly(A) signal (AATAAA) located 112 bp downstream of the *Pax6* 861 poly(A) signal (Additional file 2: Figure S1A,B). Unlike the tissue-specific expression of *Pax6*, this reverse transcript appears to be expressed ubiquitously (Additional file 2: Figure S1C). It is unclear whether this reverse orientation transcript is part of the 3’UTR for the adjacent *Elp4* gene which is transcribed in opposite orientation to *Pax6* and whose stop codon is ~ 5.5 kb away from the *Pax6* 861 poly(A) signal. Currently no non-coding RNAs map to this region.

In summary, these data indicate that the majority of mouse *Pax6* transcripts predominantly utilize a highly conserved poly(A) signal at nucleotide 861 of the 3’UTR. The remainder of this study examining the regulation of *Pax6* by microRNA will therefore focus on a 3’UTR terminating at this region. It should be noted, however, that the *Pax6* 3’UTR length is not rigidly fixed, as smaller and larger lengths (albeit less abundant) were observed. Indeed, the characterization of the original mouse *Pax6* mRNA clone [9], which focused on the largest clone obtained in that study, possessed a 3’UTR of 1008 nucleotides. Although we saw no apparent developmental differences in *Pax6* 3’UTR length we cannot rule out the possibility that *Pax6* 3’UTR length is differentially regulated in other cellular and developmental contexts.

### Identification of predicted miRNA target sites within the mouse *Pax6* 3’UTR

To identify candidate miRNAs predicted to regulate *Pax6,* we ran the mouse *Pax6* 3’UTR through an unbiased bioinformatics screen. Although functional miRNA recognition elements (MREs) have been identified within the coding regions of several genes [66–68], and MRE clusters have been identified in 5’UTRs and coding regions by Ago HITS-CLIP [69], we chose to focus on the *Pax6* 3’UTR because this is the region most commonly involved in miRNA regulation [28]. We used the prediction tool Probability of Interaction by Target Accessibility (PITA) [49] because it screens for all MREs within a mRNA sequence of interest.

**Fig. 3:**
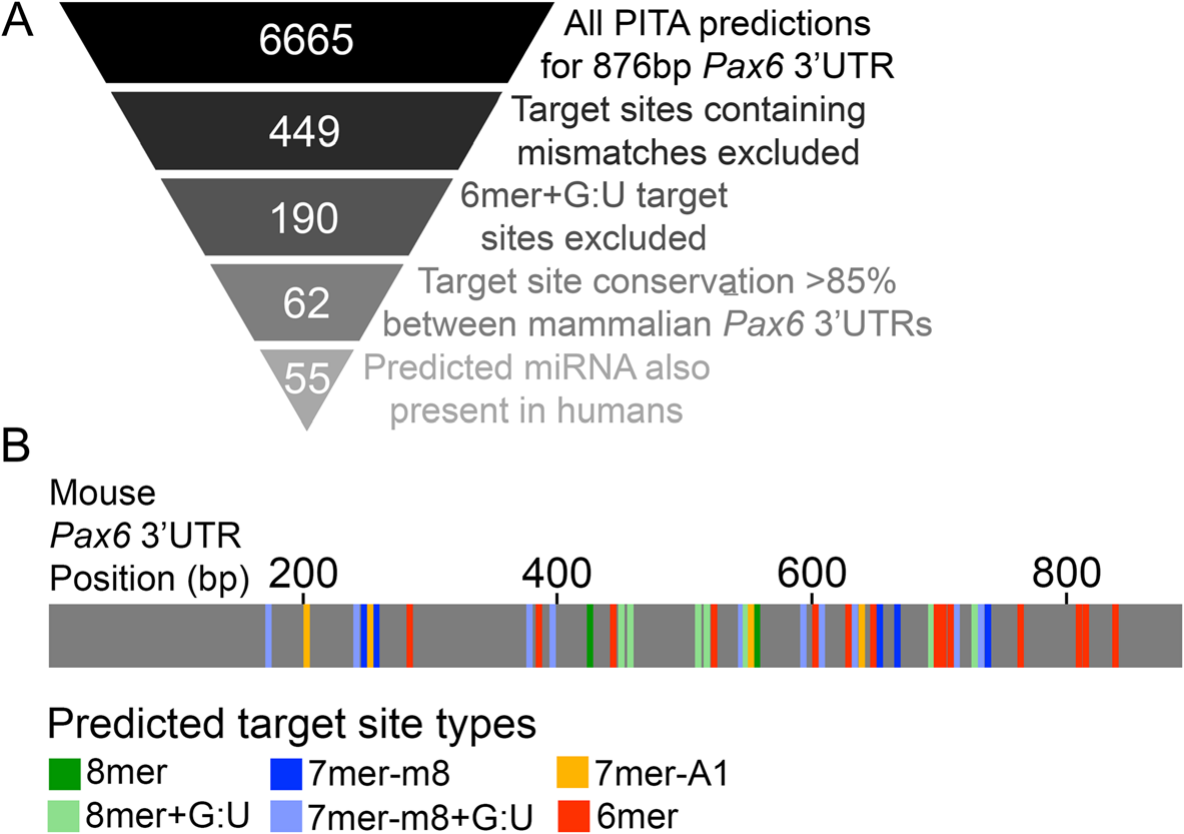
Predicted miRNA target sites in the mouse *Pax6* 3’UTR. **a** Strategy for miRNA target site selection. A total of 6665 unique hits were identified within 876 bp of the *Pax6* 3’UTR using the miRNA target site prediction tool PITA. A unique hit encapsulates a single predicted MRE-miRNA interaction. Exclusion of predicted hits containing a single mismatch between the miRNA seed and the target site in the mRNA, and predicted 6mer target sites containing a G:U wobble base pair between the miRNA and the target reduced the number of hits to 449 and then 190, respectively. Requiring a high degree of conservation between orthologous mammalian Pax6 3’UTRs (≥85%), and requiring that targeting miRNAs also be present in humans reduced the number of predicted hits to 62 and 55, respectively. Since some miRNAs share common MREs, 42 unique MREs were identified (**b**) Schematic of the mouse *Pax6* 3’UTR showing locations and target site types for the 42 unique MREs. These MREs are predicted to be targeted by 47 different miRNAs. Some miRNAs are predicted to target multiple sites within the *Pax6* 3’UTR

Our PITA screen included the four types of MREs that are matched to the miRNA 5′ end and known to be selectively conserved: 6mer, 7mer-A1, 7mer-m8 and 8mer [70]. The 6mer MRE is perfectly complementary to the “miRNA seed”, miRNA nucleotide positions 2–7. The 7mer-A1 MRE consists of a seed match with an A across from miRNA nucleotide 1, 7mer-m8 consists of a seed match with a complementary match to miRNA position 8, and the 8mer MRE consists of a seed match with both an A1 and m8. MRE types that were not considered in this analysis were offset 6mer (OS-6mer) sites, complementary to miRNA positions 3–8 [28, 71], and two site types identified by Argonaute High-Throughput Sequencing of RNA isolated by crosslinking immunoprecipitation (Ago HITS-CLIP), 6merα and G-bulge sites [72, 73]. Using PITA, 6665 unique hits, representing unique predicted miRNA-MRE interactions, were identified within 876 bp of the mouse *Pax6* 3’UTR (Fig. 3a, Additional file 3: Table S2). Though this may seem like many predictions, this is not surprising. 4526 of these predictions are MREs harboring both a mismatched pair and G:U pair between the miRNA and MRE. Additionally, 1187 of these predictions are 6mer MREs harboring a single mismatch between the miRNA and MRE. Given that the PITA miRNA database contains 491 mouse miRNAs, the prediction of these types of MREs by chance is high.

In order to focus our analysis on those miRNAs most likely to regulate *Pax6*, we next screened the 6665 *Pax6* hits against a set of hierarchical criteria (Fig. 3a). Mismatches between a miRNA and MRE are less likely to be associated with functional target sites [74] and are rarely under selection to be evolutionarily conserved [71]; these predicted MREs were excluded, narrowing the total number of predicted hits to 449. miRNA-target interactions containing single G:U “wobble” pairs can function effectively in downregulation endogenously [75] and in reporter screens [40], and simulations of Ago-miRNA:mRNA complexes and Ago HITS-CLIP data suggest that G:U wobbles can be tolerated [69, 76]. However, though 7 and 8mer MREs containing single G:U pairs functioned in downregulation of reporters in *Drosophila* in vivo, 6mer MREs containing single G:U pairs were non-functional [74]. For this reason, we chose to exclude 6mer + G:U MREs from consideration, which further narrowed the number of predicted hits to 191.

Target sites that show high conservation between orthologous 3’UTRs are more likely to be functional [70, 77]. Using UCSC Genome Browser [78] alignments, a total of 62 predicted MREs were found to be conserved in ≥85% of orthologous *Pax6* 3’UTR sequences from placental mammals. Finally, as MREs for miRNAs that are broadly conserved are more likely to be functionally conserved [71]. Using miRBase [79], we identified and excluded miRNAs that are rodent-specific and retained miRNAs that are found in humans. This reduced the number of unique hits to 55. Given that some MREs are predicted to be targeted by multiple miRNAs, and some miRNAs are predicted to target multiple MREs within the *Pax6* 3’UTR, we identified 47 candidate miRNAs of interest and 42 candidate MREs (Fig. 3a, Additional file 3: Table S2 listed as “Primary Candidate” under “Reason Selected”). 12 of these MREs are predicted to be targeted by multiple miRNAs and 25 of the identified miRNAs are predicted to target multiple MREs within the *Pax6* 3’UTR. Of these 25 miRNAs, 8 have multiple MREs that are conserved in > 85% of placental mammal alignments (Additional file 1: Table S1). In summary, we have identified 47 MREs within the *Pax6* 3’UTR that satisfy our selection criteria as strong candidates for miRNA interaction. Some of the MREs and the miRNAs that target them exhibit redundancy which should be considered for any functional analyses. It should be recognized that although these 47 MREs represent strong candidates for miRNA interaction, it does not necessarily mean that they are functional in vivo, nor does it mean that other MREs that did not meet our criteria are not functional. This analysis, however, does provide a starting point for studying *Pax6* regulation by miRNA.

### Expression profiling of miRNAs predicted to target the mouse *Pax6* 3’UTR

**Fig. 4:**
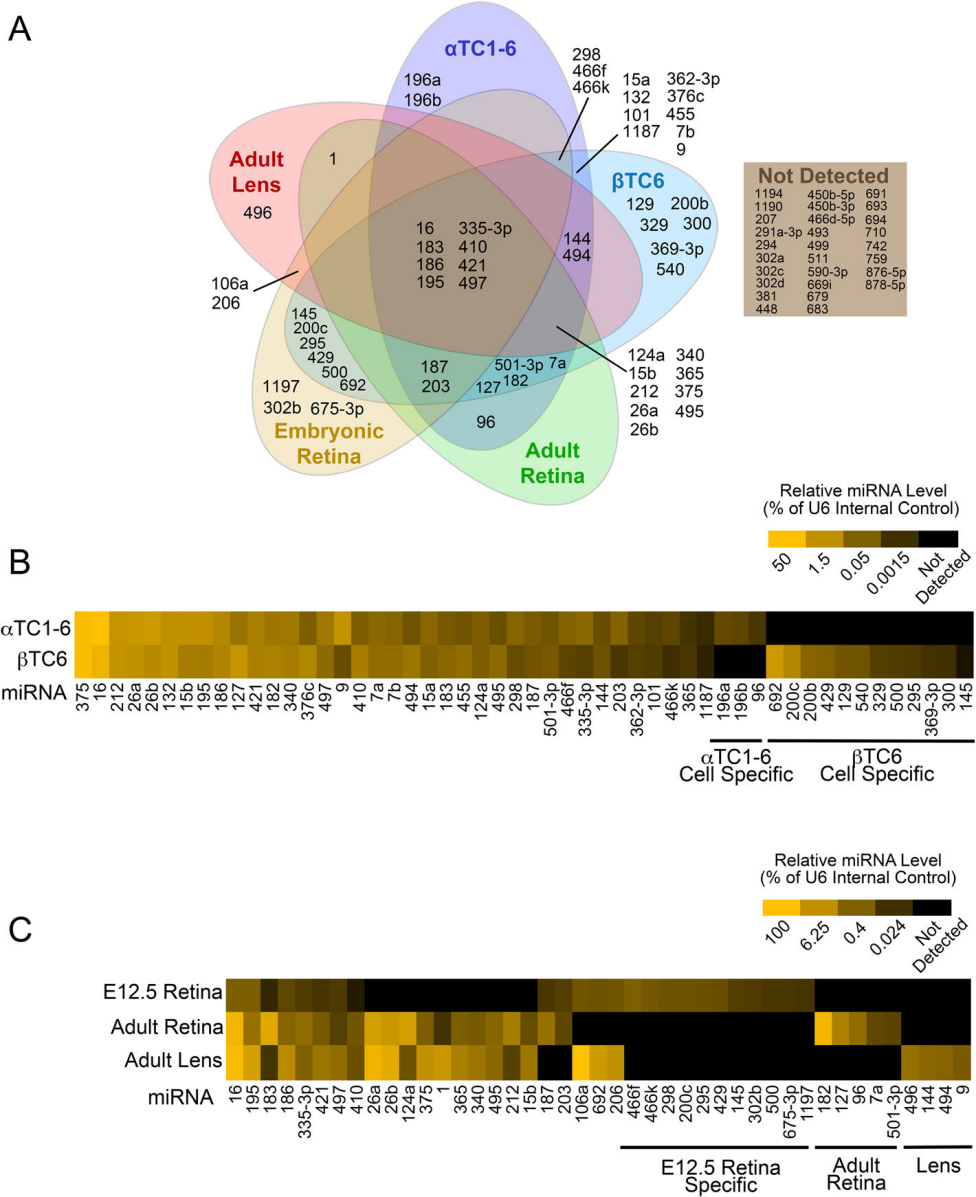
Expression profile of miRNAs predicted to target the mouse *Pax6* 3’UTR. **a** Expression profile of miRNAs having predicted target sites in the mouse *Pax6* 3’UTR in various *Pax6*-expressing cells and tissues. miRNAs assayed for include those identified by our prediction analysis (Fig. 3a) as well as others, as described in the results. miRNAs were assayed for using TaqMan multiplex qPCR array cards. Data represents a total of 3 replicates per tissue or cell type, with a miRNA considered to be expressed only if the cycle threshold was less than 40 for all three replicates. **b** Relative miRNA level in mouse cultured pancreatic α cell line, αTC1–6 and a cultured β cell line, βTC6. **c** Relative miRNA level in mouse E12.5 retina, adult retina, and adult lens. Heat map indicates relative miRNA expression as a percentage of an internal control, snRNA U6. Data represents the geometric mean of three independent samples. Note the scale differences between the two heat maps

We next sought to determine the expression profile of the miRNAs identified above that are predicted to target to *Pax6* 3’UTR. Our analysis focused on Pax6-expressing ocular tissues (embryonic day (E) 12.5 retina, adult retina and adult lens) and on the SV40-induced cell lines α-TC1–6 and β-TC6 which serve as in vitro models for endocrine pancreas α and β cells, respectively [80, 81]. Both cell lines endogenously express Pax6 [82, 83]. MiRNA expression was detected using TaqMan multiplex quantitative PCR (qPCR) arrays (Applied Biosystems). Extra wells available on the arrays allowed for the detection of additional miRNAs predicted to target less well conserved 7mer and 8mer MREs for expression profiling. 59 of the miRNAs examined were detected in at least one of the profiled cells or tissues, while 28 of the miRNAs did not have detectable expression in any of the tissues/cells examined (Fig. 4a).

Our data revealed distinct tissue/cell-specific miRNA expression patterns arising from overlapping and non-overlapping miRNA expression (Fig. 4a). All tissues/cells except for the adult retina had at least one uniquely expressed miRNA. These distinct expression patterns may allow for cell-type specific optimization of Pax6 expression through cooperative miRNA regulation.

The endocrine pancreas α and β cell lines for the most part had very similar miRNA expression patterns (Fig 4b). miR-375 was among the most highly expressed miRNAs in both α and β cells, consistent with previous expression studies which have also reported similar high miR-375 expression levels in endogenous human α and β cells [84]. miR-375, in addition to miR-16, 26a, 26b, 124 and 127, has been identified as an abundant miRNA in MIN6 and αTC1 cells [85], and human islets [86]. Additionally, many miRNAs identified as islet-enriched relative to acinar cells (miR-7, 96, 127, 132, 183, 335) were identified in αTC1–6 or βTC6 cells by our analysis [87]. A smaller subset of α-specific and β-specific miRNAs was also observed. Some of these (miR-129, 145, 200b, 200c, 369-3p, 429), have previously been shown to be more abundantly expressed in β relative to α cells [84, 85], and serve to validate the veracity of the Taqman array cards. It should be noted that miR-96, a miRNA that we detected at low levels in αTC1–6 but not βTC6, was found to be expressed more abundantly in human β relative to α cells [84], possibly demonstrating a difference between the mouse and human endocrine pancreas cells.

Many of the miRNAs profiled displayed differential patterns of expression between E12.5 retina, adult retina, and adult lens (Fig. 4c), similar to observations from microarray analyses finding differential expression of miRNAs in developing retina versus adult retina [88], and between adult ocular tissues [89]. For example, miR-124, 182, 183 and 96 were previously identified as adult retina-enriched miRNAs [90, 91] and these findings have been validated by our analysis. These results suggest that some of the differentially-expressed ocular miRNAs may have tissue-specific functions, potentially through the regulation of *Pax6*. Interestingly, the adult retina had an expression profile that was more similar to adult lens than to the E12.5 retina, which had the fewest miRNAs of the cells/tissue examined (Fig. 4c). Additionally, of the miRNAs profiled, E12.5 showed overall lower miRNA expression than adult retina or lens (Additional file 4: Figure S2 C, D, E). This is not surprising given that miRNA abundance increases with cellular differentiation [92, 93] and during development [94–97], and fits with the proposal that miRNAs function primarily during differentiation and the maintenance of tissue identity [98, 99]. It should be noted that the expression level of some miRNAs in some tissues is highly variable, and this variability is particularly apparent for less abundant miRNAs. Notably, E12.5 retina-expressed miRNAs, which exhibit low levels of miRNA expression relative to other tissue and cell types, were especially variable in their level of expression (Additional file 4: Figure S2).

### Characterization of a *Pax6* miR-code in αTC1–6 cells

**Fig. 5:**
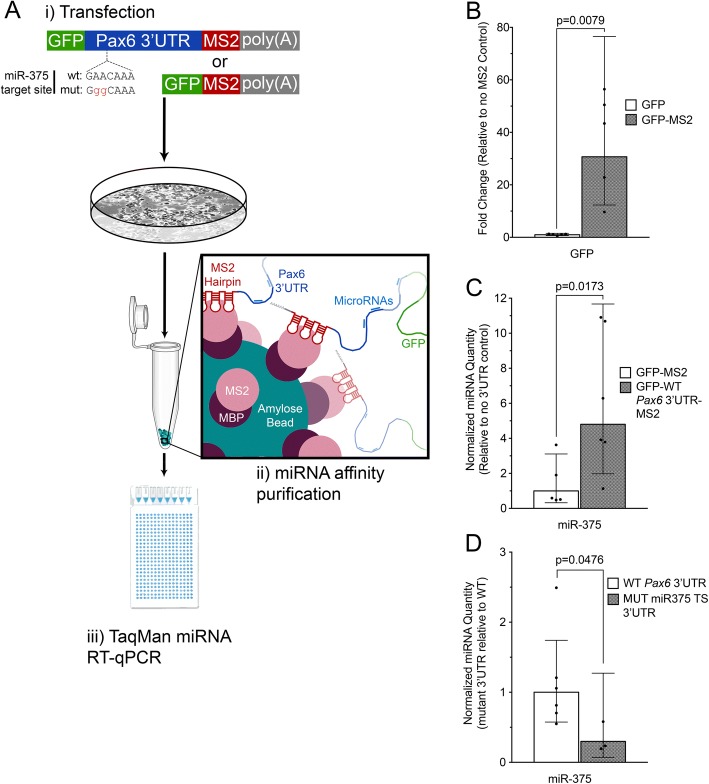
miTRAP as a strategy to purify *Pax6* 3’UTR-associated miRNAs. **a** Schematic of the *Pax6* 3’UTR affinity purification approach. (i) Plasmid vectors expressing GFP tagged with the MS2 RNA sequence motif followed by the SV40 polyadenylation signal are introduced into pancreatic αTC1–6 cells via transient transfection. (ii) MS2 coat protein fused to maltose binding (MS2-MBP) is used to purify GFP transcripts with bound miRNAs from αTC1–6 cell lysate. (iii) Real-time quantitative PCR (RT-qPCR) is used to detect GFP transcript and bound miRNAs. Schematic of the *Pax6* 3’UTR shows the location of the highly conserved miR-375 target site located at 3’UTR position 201 and miR-375 target site mutation. **b** Validation of the MS2-mediated affinity purification strategy by RT-qPCR quantification of GFP transcripts with and without the MS2 RNA motif. Fold change was calculated using Pfaffl’s method [61]. qPCR results for GFP with the MS2 motif (grey bar) were expressed relative to data without the MS2 motif (unfilled bar). Data represents 5 independent samples, *p* = 0.0079. **c** Affinity purification of miR-375 with the *Pax6* 3’UTR in αTC1–6 cells using TaqMan individual qPCR assays. Normalized relative quantity was calculated using Pfaffl’s method, and a normalized relative quantity greater than 1 indicates that more target miRNA is purified with the *Pax6* 3’UTR (grey bar) than the control lacking a *Pax6* 3’UTR (unfilled bar). Data represents six independent samples, *p* = 0.013. **d** Disruption of miR-375 binding to the *Pax6* 3’UTR following mutation of the miR-375 target site. Target miR-375 values were normalized to GFP as a reference gene, then normalized values for the mutant *Pax6* 3’UTR samples (grey bar), and are presented relative to the wt 3’UTR (unfilled bar). Data represents six independent wt 3’UTR samples and three miR-375 target site mutant 3’UTR samples, *p* = 0.0476. Error bars represent 95% confidence intervals, and *p*-values were calculated using the Mann Whitney test. Note scale bar differences between the graphs

While the miRNA expression profiles generated above provide valuable information about potential cell and tissue-specific *Pax6* regulation, they do not provide information about whether any of the miRNAs actually interact with the *Pax6* 3’UTR. To address this, we utilized the MS2 RNA stem-loop sequence fused to exogenously-expressed mouse *Pax6* 3’UTR as “bait” RNA to co-purify bound miRNAs in an approach called miRNA Trapping by in vitro RNA Affinity Purification (miTRAP) [100]. The MS2 RNA stem-loop structure has been shown to interact specifically with the MS2 bacteriophage coat protein [101], and this interaction can be harnessed for RNA affinity purification techniques [57]. miTRAP was originally used to identify miRNAs, miR-1 and miR-133, associated with the 3’UTR of Hand2 in primary cardiomyocytes [37], and has since been used to identify miRNAs associated with the 3’UTRs of MYC and ZEB2 [100], and with the long intergenic non-coding RNA (lincRNA) p21 [102]. Our approach (summarized in Fig. 5) utilized a reporter construct in which GFP was engineered to carry a modified *Pax6* 3’UTR containing three MS2 binding motifs positioned immediately 5′ of the poly(A) signal. αTC1–6 cells were transiently transfected with MS2-tagged GFP constructs, and pull-down products were amplified using TaqMan multiplex qPCR and normalized to GFP mRNA transcripts amplified using SYBR green qPCR.

**Fig. 6:**
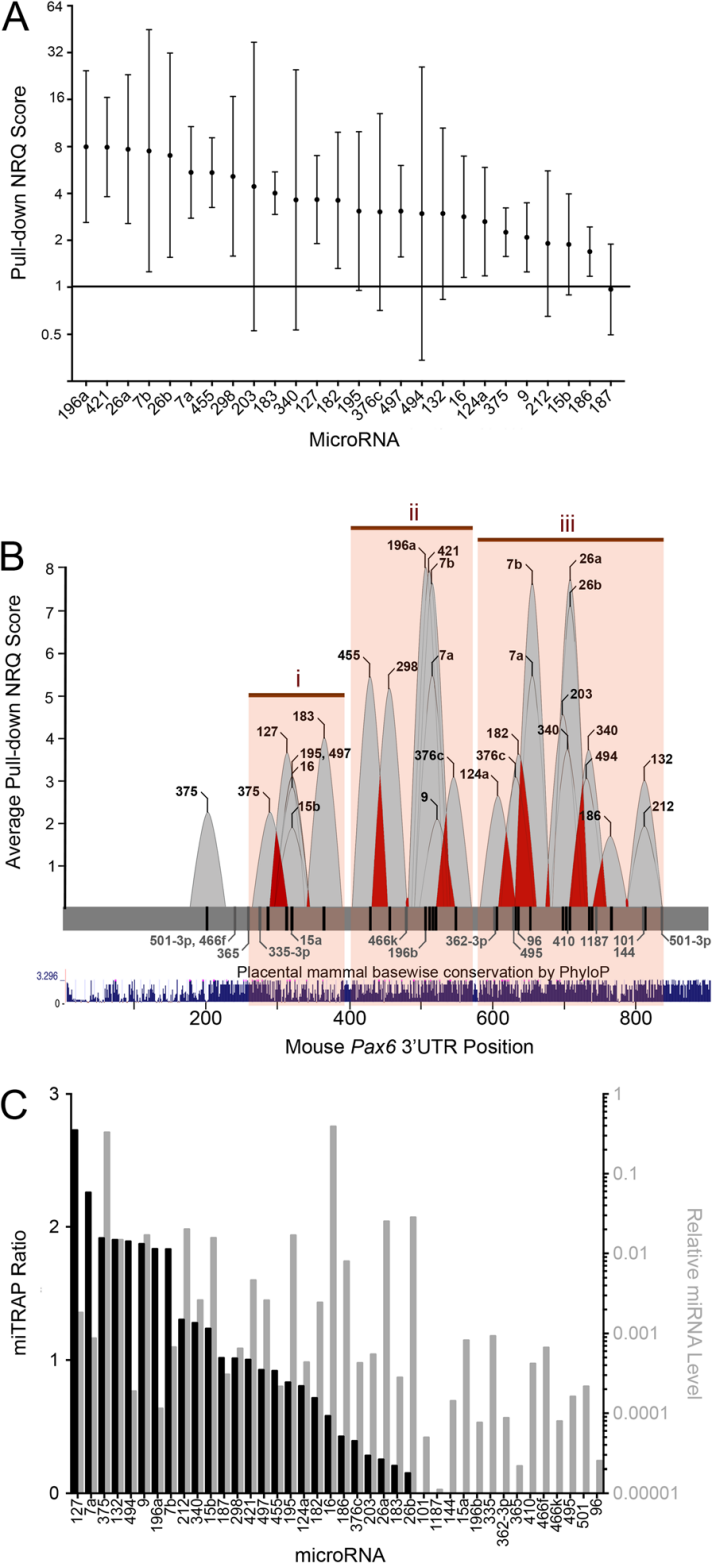
Characterization of miRNAs bound to the *Pax6* 3’UTR in pancreatic α cells. **a** Identification of miRNAs associated with the *Pax6* 3’UTR in αTC1–6 cells using TaqMan multiplex qPCR arrays. Normalized relative quantity (NRQ) greater than 1 indicates more target miRNA was purified with the *Pax6* 3’UTR than the control lacking a *Pax6* 3’UTR. Some miRNAs expressed in α cells that also have predicted MREs in the *Pax6* 3’UTR did not associate with the *Pax6* 3’UTR-containing transcript: miR-101, 1187, 144, 15a, 196b, 335-3p, 362-3p, 365, 410, 466f, 466 k, 495, 501–3p, 96. Results represent four independent experiments. Geometric mean +/− 95% confidence intervals are shown. **b** Landscape of *Pax6* 3’UTR miRNA interaction in αTC1–6 cells. Average NRQ value for each interacting miRNA is indicated as a peak at the 3’UTR position(s) of the predicted MRE(s). Predicted MREs for interacting miRNAs are labeled in black, and non-interacting miRNAs in grey. Each peak has a 25-nucleotide width on either side of the MRE position. Overlap between interaction peaks spaced 8–50 nucleotides apart is indicated in red, and these interacting miRNAs may be capable of mediating cooperative regulation of *Pax6.* MREs for *Pax6* 3’UTR-interacting miRNAs were found to cluster into three regions, i-iii (orange boxes), located approximately at nucleotide positions 250–350, 420–550 and 600–810. Conservation of the *Pax6* 3’UTR sequence between orthologous placental mammal sequences is shown for the 876-nucleotide length. Secondary, poorly conserved MREs for interacting miRNAs having well conserved predicted MREs are not shown. **c** miRNA interaction with the *Pax6* 3’UTR is not directly related to miRNA abundance in αTC1–6 cells. miTRAP ratio and relative miRNA level for αTC1–6-interacting miRNAs is shown. miTRAP ratio was calculated by dividing the relative abundance of each miRNA with the *Pax6* 3’UTR by the relative abundance in αTC1–6 cells. Larger miTRAP values indicated greater enrichment of the miRNA with the *Pax6* 3’UTR relative to cellular abundance

Three steps were taken to demonstrate the specificity of our miTRAP approach. First, to show that MS2-containing mRNAs were selectively enriched, we compared the pull-down of GFP constructs with or without the MS2 binding motif. GFP reporter transcripts harboring the 3xMS2 motif exhibited a 30-fold increase in enrichment compared to those lacking the MS2 binding motif (Fig. 5b), demonstrating robustness of the pull-down process. Next, we examined the binding of the *Pax6* 3’UTR with a known interactor, miR-375 [103, 104]. Approximately five-fold more miR-375 was pulled-down with GFP constructs carrying the wild type *Pax6* 3’UTR compared to those with an SV40 3’UTR (Fig. 6b), indicating *Pax6*–3’UTR mediated enrichment. To determine the specificity of this interaction, pull-down experiments were performed using constructs carrying mutations that disrupt a previously described 7mer-A1 MRE for miR-375 at position 201 of the *Pax6* 3’UTR [103, 104]. Mutation of this MRE resulted in 4-fold reduced binding of miR-375 compared to the wild type *Pax6* 3’UTR (Fig. 5c), indicating its requirement for interaction with miR-375. A second miR-375 MRE (6mer site) is also present at position 288 of the *Pax6* 3’UTR, which may explain why binding is not completely lost. It is therefore possible that disruption of both MREs would further reduce miR-375 binding.

The miTRAP approach was next used in conjunction with TaqMan multiplex qPCR arrays to identify the miRNAs that physically interact with the *Pax6* 3’UTR. Relative abundance of target miRNA transcripts associated with the *Pax6* 3’UTR was normalized to GFP and expressed relative to the SV40 3’UTR control using Pfaffl’s method to produce a normalized relative quantity (NRQ) value [61]. An NRQ value greater than 1 indicates that more of a given miRNA was purified with the *Pax6* 3’UTR than with the control 3’UTR. Of the 40 miRNAs having MREs in the *Pax6* 3’UTR that were also expressed in cultured αTC1–6 cells (Fig. 4), 25 were found to have NRQs greater than 1 (Fig. 6a, summarized in Table 1). Eight of these 25 miRNAs, however, had 95% confidence intervals extending below a NRQ of 1, indicating lower confidence that they bind the *Pax6* 3’UTR preferentially over the control SV40 3’UTR. miR-187 was pulled-down equally using MS2-tagged reporters with and without the *Pax6* 3’UTR suggesting non-specific interaction, while 14 of the 40 miRNAs assayed were undetectable in MS2-tagged *Pax6* 3’UTR reporter pull-downs. As such, we can be reasonably confident that 17 of the assayed miRNAs interact with the *Pax6* 3’UTR.

**Table 1:** Summary of miTRAP interactions in αTC1–6 cells

miTRAP Results		MRE Type	miRNA
Interaction (62.5%, 25/40)	High Confidence (42.5%, 17.40)	8mer	455
		7mer-m8	*127*^a^, **7a**^b^, **7b**^b^, **196a**^a^, 298^a^, 421^ab^, **497**^a^, 124a^a^, *16*^a^, 26a^a^, 26b^a^
		7mer-A1	**375**^b^, 182^a^
		6mer	**9**^b^, 186, *183*
	Low Confidence (20%, 8/40)	8mer	494^a^
		7mer-m8	132^ab^, *15b*^a^, *195*^a^, 376c^a^
		6mer	212^b^, **340**^+++^, **203**
Non-specific Interaction (2.5%, 1/40)		7mer-m8	*187* ^a^
No Interaction (35%, 14/40)		8mer	*335-3p* ^a^
		7mer-m8	1187^a^, *15a*^a^, *196b*^a^, **365**^b^, *466k*, 501–3p^b^
		7mer-A1	96^ab^
		6mer	101, 144, **362-3p**, **410**, 466f, **495**

miRNAs identified in present study as well as by TargetScan, miRanda, MicroCosm, or DIANA-microT are depicted in bold

^a^Predicted MRE-miRNA seed interaction contains a G:U pair

^b^indicates miRNAs with additional, weaker MREs in the *Pax6* 3’UTR

miRNAs having MREs that are conserved in less than 85% of placental mammal *Pax6* 3’UTR sequence alignments are depicted in italics

A potential criticism of the miTRAP approach is that it could be biased toward pulling-down abundantly expressed miRNAs while low-expressed miRNA interactors might be missed [100]. We observed that this was not the case. Eleven of the 17 miRNAs pulled-down with the *Pax6* 3’UTR were expressed at levels less than 0.5% relative to the U6 internal control (miR-124a, 127, 182, 183, 196a, 298, 421, 455, 497, 7a and 7b; Additional file 4: Figure S2A). Notably, miR-196a, which was expressed at 0.01% relative to U6, was one of the more abundantly enriched miRNAs pulled-down with the *Pax6* 3’UTR, having a NRQ of 8.0 (Fig. 6a). In contrast, several miRNAs with relative expression levels higher than miR-196a in αTC1–6 (e.g. miR-15a, 144, 335, 410, 466f, 495, 501), were not pulled-down at all (Fig. 6c). Furthermore, miR-16 and miR-375, which were by far the most abundantly expressed miRNAs in αTC1–6 cells, had NRQ values (2.8 and 2.3, respectively) lower than that of miR-196a (Fig. 6a). It is important to recognize that while our miTRAP data revealed miRNAs with the capacity to interact with the *Pax6* 3’UTR, it may not necessarily reflect miRNA binding to the endogenous *Pax6* 3’UTR. Bait mRNAs used for our experiments were driven from overexpression plasmids and harbour elements such as the MS2 binding domain, which could potentially influence miRNA binding. To address some of these issues and confirm our data, other miRNA:mRNA interaction approaches could be taken [105].

Individual miRNAs tend to have a modest impact on the protein output from targeted transcripts [32] and knock-out of individual miRNA genes or clusters are often not associated with overt phenotypes [106–114]. One explanation for these findings is that regulation of target transcripts by miRNAs may involve the collective action of multiple miRNAs. In support for this, multiple MREs can have a cooperative impact on target transcript destabilization [35] and genetic reporter protein synthesis [36] when MREs are approximately 8–50 nucleotides apart. We therefore probed the MRE positional code for the miRNAs identified by miTRAP to address the potential for cooperative miRNA regulation of *Pax6*. MREs for *Pax6* 3’UTR-interacting miRNAs were found to cluster into three regions located approximately at nucleotide positions 250–350, 420–550 and 600–810 (Fig. 6b) (note that some of these miRNAs share common or overlapping MREs, and would thus be incapable of acting cooperatively). Interestingly, miRNAs with the highest NRQ values clustered in the 3′-half of the 3’UTR with regions ii and iii. Additionally, the *Pax6* 3’UTR shows poor conservation of the 5′ end between orthologous placental mammal *Pax6* 3’UTR sequences. Previous work has shown that miRNA repression is preferential at the ends of the 3’UTR and poor in the middle [35]. This study, however, was conducted using long (> 1300 nt) 3’UTRs and assayed for repression of the target mRNA, so it is difficult to make any direct comparisons to our study.

Association of miRNAs to the *Pax6* 3’UTR does not provide direct evidence that multiple MREs are contributing to cooperative binding and regulation, or that bound miRNAs are acting to regulate transcript stability or protein synthesis. It is not clear from our miTRAP experiments whether multiple different miRNAs are binding a single transcript simultaneously and to what extent multiple MREs for individual miRNAs are contributing to binding. A sequential pulldown approach using biotinylated miRNA could be used to address simultaneous binding of multiple different miRNAs [37]. Additionally, performing miTRAP using the *Pax6* 3’UTR with mutations introduced into individual MREs, similar to the approach used in Fig. 4e, could be used to determine whether binding of miRNAs to un-mutated closely spaced MREs are also impacted, and to address the relative contribution of multiple MREs for the same miRNA. Genetic reporter assays could be used to address the functionality of MREs that participate in binding α cell-expressed miRNAs, and could also be used to address cooperative regulation through closely-spaced MREs found to bind miRNAs. Ultimately, generation of mice harbouring mutations in multiple MREs found to bind α cell-expressed miRNAs could be used to address whether regulation of *Pax6* by these miRNAs plays an important role and α cell, and more generally endocrine pancreas, development and function.

Several of the miRNAs identified in our study by miTRAP have been implicated in processes relevant to Pax6 function in endocrine pancreas development. Two miRNAs that have previously been implicated in the regulation of *Pax6*, miR-7 and 375, may be critical for proper development of hormone-producing cells. Knockdown of miR-7 in embryonic mouse pancreas explants increased the proportion of insulin and glucagon-positive cells, and in vivo overexpression had an opposing effect, presumably through regulation of *Pax6* [46]. Knockdown of miR-375 during zebrafish embryonic development resulted in disorganized development of the pancreatic islet [115], and miR-375-null mice have reduced insulin positive cells per pancreas with a corresponding increase in the number of glucagon positive cells [109]. Three miRNAs identified in our miTRAP experiments that have not previously been implicated as *Pax6* regulators, miR-15, 16 and 195, result in reduced insulin and glucagon positive cells when overexpressed in developing pancreatic buds [116].

Several miRNAs identified using miTRAP are also associated with processes relevant to Pax6 function in glucose metabolism and endocrine pancreas gene transcription raising the possibility that they function, in part, through regulation of *Pax6*. For instance, several *Pax6* 3’UTR interacting miRNAs (miR-124a, 132, 212, 494, and 9) are upregulated and miR-375 is downregulated in mouse β cells and pancreatic islets under hyperglycemic conditions [117–119]. Overexpression of *Pax6* 3’UTR interacting miRNAs miR-124a, 375 and 9 decreases the secretory capacity of β cell lines [85, 119, 120] and glucose tolerance improves in mice harboring genetic deletion of the miR-7a-2 gene [108]. Knockdown of miR-7 also increases insulin promoter activity in cultured β cell lines [121] and insulin mRNA from pancreas explants [46]. Together these findings support the idea that *Pax6* participates in a genetic network with the identified targets of these miRNAs to impact glucose-stimulated insulin secretion.

To date, only miR-375 and miR-7 have been investigated in the context of α cell hormone production and secretory function. *miR-375*-null mice had elevated plasma glucagon levels and glucagon secretion from isolated islets relative to controls [109] and miR-7 knockdown and overexpression during mouse pancreatic development elevates and represses glucagon mRNA expression, respectively [46]. How and whether the other miRNAs identified by miTRAP in α-TC1–6 cells contribute to α cell function remains to be determined.

## Conclusions

Our work provides a comprehensive analysis of the *Pax6* 3’UTR and description of potential miRNA recognition elements. We have identified cell type-specific miRNA cohorts or “miR-codes” that form 3’UTR interaction clusters where cooperative regulation of *Pax6* may occur. As proper PAX6 function is highly sensitive to its dosage, miRNA post-transcriptional regulation is predicted to play an important role in maintaining correct PAX6 levels. Our work reveals a regulatory landscape upon which the role of miRNAs on the developmental control, fine-tuning and/or homeostasis of PAX6 can be further investigated.

## Additional files

Additional file 1: **Table S1.** microRNAs identified as candidates to regulate *Pax6.* miRNAs predicted to target 876 bp of the *Pax6* 3’UTR that were selected for inclusion on TaqMan microfluidic qPCR cards. Information about the MREs and rationale for selection are included. (XLSX 17 kb)

Additional file 2: **Figure S1.** Characterization of a reverse orientation transcript terminating directly adjacent to the Pax6 mRNA 3′ terminus (DOCX 423 kb)

Additional file 3: **Table S2.** PITA predictions for 876 nucleotides of *Pax6* 3’UTR. Full list of MREs predicted by PITA for 876 bp of the *Pax6* 3’UTR, and MRE exclusion criteria. (XLSX 135 kb)

Additional file 4: **Figure S2.** Relative miRNA level in aTC1–6, bTC6, E12.5 retina, adult retina, adult lens. Expression level of miRNAs from TaqMan microfluidic qPCR cards relative to U6 in αTC1–6 cells, βTC6 cells, mouse E12.5 retina, adult retina and adult lens. (DOCX 398 kb)

## Abbreviations

3’UTR: 3′ untranslated region
miRNA: microRNA
MRE: miRNA recognition element
mRNA: Messenger RNA
NRQ: Normalized relative quantity
poly(A): Polyadenylation
SVZ: Subventricular zone
