# Supplementary material for: Mapping the Pax6 3’ untranslated region microRNA regulatory landscape
Source: BMC Genomics. 2018 Nov 15;19:820. doi: 10.1186/s12864-018-5212-x (PMC6238409; doi:10.1186/s12864-018-5212-x)
Supplement: Supplementary file 2 — Figure S1. Characterization of a reverse orientation transcript terminating directly adjacent to the Pax6 mRNA 3′ terminus (DOCX 423 kb) [file 12864_2018_5212_MOESM2_ESM.docx]

##
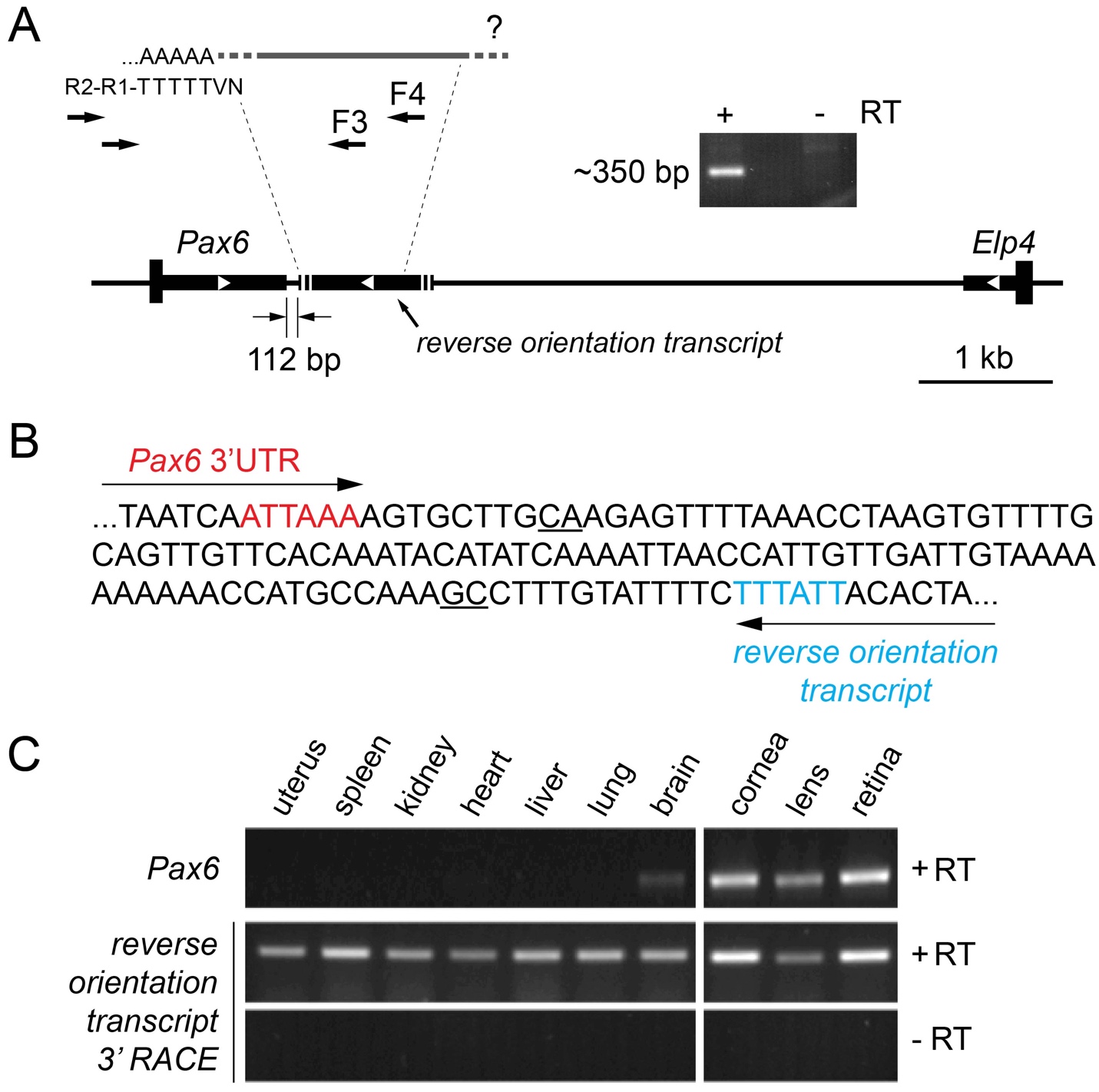


## Supplementary Figure 1. Characterization of a reverse orientation transcript terminating directly adjacent to the *Pax6* mRNA 3’ terminus

(A) 3’ RACE amplification strategy used identify a reverse orientation transcript positioned close to the *Pax6* mRNA 3’ end. The wider bars in the schematic diagram represent coding regions and the narrower bars represent 3’UTRs. Nested primers F3 and F4 were used in combination with reverse primers (R1, R2) built into the poly-T primer to amplify a robust ~350 base pair product. (B) Sequencing of the reverse orientation transcript shows that it terminates at a “GC” (underlined). The reverse transcript appears to utilize a polyA signal (blue font) that is 112 bp away from the end of the polyA signal at position 861 of the *Pax6* 3’UTR (red font). (C) 3’RACE of the reverse orientation transcript (bottom 2 panels) and reverse transcription of *Pax6* (top panel) showing widespread expression of the revere orientation transcript in contrast to the tissue specific expression of *Pax6*.
