# Supplementary material for: Mapping the Pax6 3’ untranslated region microRNA regulatory landscape
Source: BMC Genomics. 2018 Nov 15;19:820. doi: 10.1186/s12864-018-5212-x (PMC6238409; doi:10.1186/s12864-018-5212-x)
Supplement: Supplementary file 4 — Figure S2. Relative miRNA level in aTC1–6, bTC6, E12.5 retina, adult retina, adult lens. Expression level of miRNAs from TaqMan microfluidic qPCR cards relative to U6 in αTC1–6 cells, βTC6 cells, mouse E12.5 retina, adult retina and adult lens. (DOCX 398 kb) [file 12864_2018_5212_MOESM4_ESM.docx]

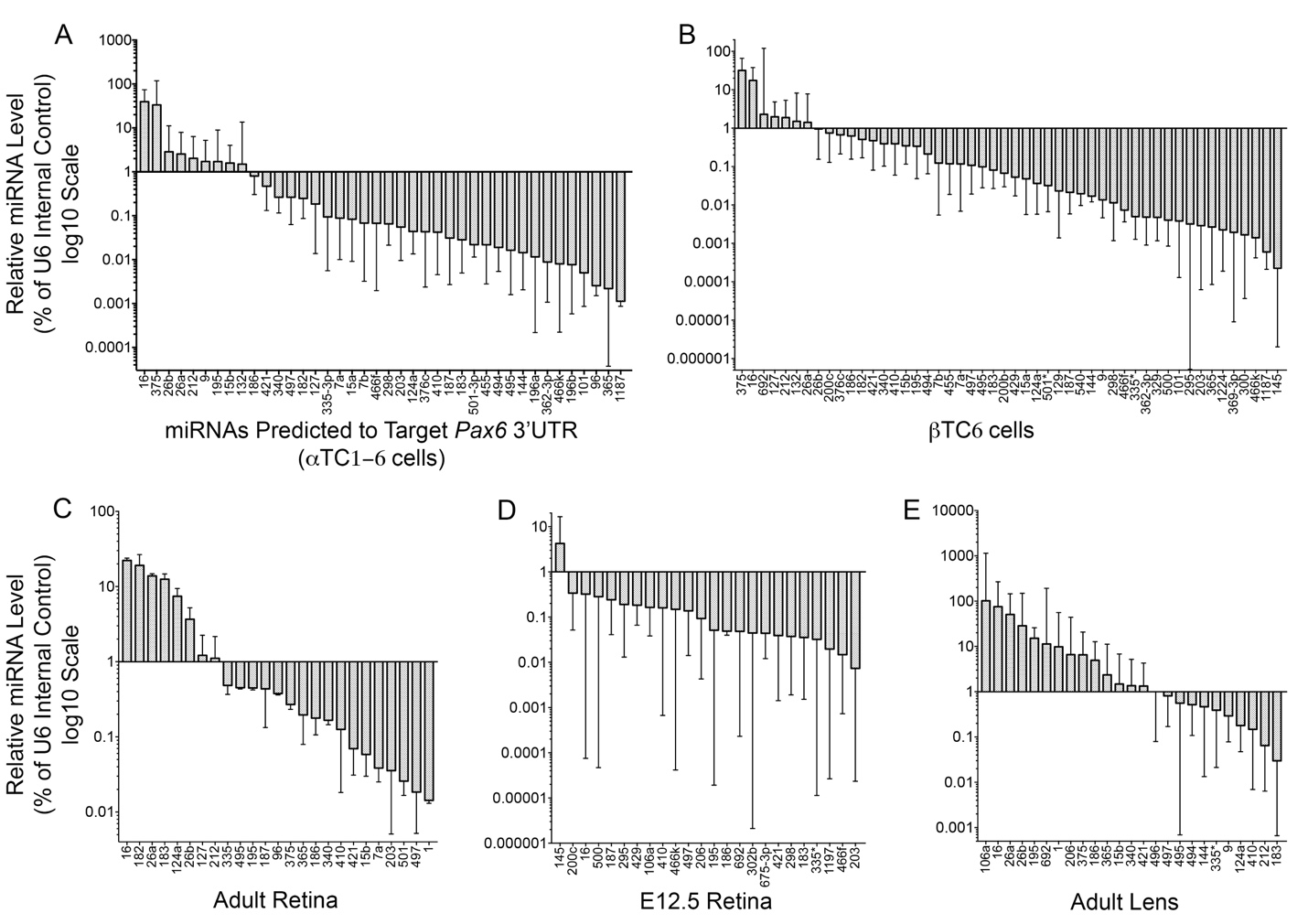


## Supplementary Figure 1

(A) Relative miRNA level in mouse cultured pancreatic α cell line, α-TC1-6, (B) a cultured β cell line, βTC6, (C) adult retina, (D) E12.5 retina and (E) adult lens expressed as a percent of snRNA U6 internal control. Data represents the geometric mean of three independent samples and error bars represent 95% confidence intervals. Data is represented using log10 scale; note scale differences between graphs.
